# Complete Genome Sequence of *Edwardsiella ictaluri* Isolate RUSVM-1 Recovered from Nile Tilapia (*Oreochromis niloticus*) in the Western Hemisphere

**DOI:** 10.1128/genomeA.00390-17

**Published:** 2017-06-15

**Authors:** Stephen R. Reichley, Geoffrey C. Waldbieser, Esteban Soto, Mark L. Lawrence, Matt J. Griffin

**Affiliations:** a College of Veterinary Medicine, Mississippi State University, Starkville, Mississippi, USA; b Aquatic Research and Diagnostic Laboratory, Thad Cochran National Warmwater Aquaculture Center, Stoneville, Mississippi, USA; c USDA-ARS Warmwater Aquaculture Research Unit, Thad Cochran National Warmwater Aquaculture Center, Stoneville, Mississippi, USA; d Department of Medicine and Epidemiology, School of Veterinary Medicine, University of California, Davis, Davis, California, USA

## Abstract

*Edwardsiella ictaluri* is a Gram-negative bacillus that has recently been implicated in disease outbreaks in tilapia and zebrafish. We report here the complete and annotated genome sequence of an isolate from a Nile tilapia (*Oreochromis niloticus*), which contains a chromosome of 3,630,639 bp and two plasmids.

## GENOME ANNOUNCEMENT

Enteric septicemia of catfish (ESC) was first detected in the late 1970s, predominantly in pond-reared fingerling channel catfish (*Ictalurus punctatus*) (1). The causative agent of ESC was later determined to be *Edwardsiella ictaluri*, a Gram-negative bacillus member of *Enterobacteriaceae* (2). Since its emergence, ESC has become a major pathogen of farm-raised catfish, causing significant economic losses (3). Although traditionally associated with mortality in catfish, the bacterium has recently been implicated in disease outbreaks in tilapia and zebrafish (4, 5). This suggests a broader distribution of *E. ictaluri* than previously thought.

Genomic DNA sequence was produced on a MinION sequencer with the rapid sequencing kit and an R9 flow cell (Oxford Nanopore Technologies, Oxford, England, UK). Raw data (754 Mb) were error-corrected and trimmed to a total of 129 Mb in 8,795 sequences (average length, 14,660 bp) and assembled using Canu version 1.3 (6). The assembled genome contig contained an average 32× read coverage per base. The linear contig was circularized and relinearized at a position 1 million bases from the start position, and Nanopore sequences were mapped to the contig using BWA version 0.7.15-r1140 (7) and visualized using Integrative Genomics Viewer (IGV) (8) to validate contiguity. Paired Illumina sequences were mapped to the genome contig to a minimum coverage depth of 218×, and then assembly errors were corrected through four iterations of Pilon version 1.21 (9).

The circularized and completed genome was submitted to the National Center for Biotechnology Information (NCBI) Prokaryotic Genome Annotation Pipeline (PGAP) for annotation and submission to GenBank. The genome was also submitted for Rapid Annotations using Subsystems Technology (RAST) analysis (10, 11), employing the Glimmer option. Average nucleotide identity (ANI) (12) and digital DNA-DNA hybridization (dDDH) (13) estimations were determined using online calculators (ANI, http://enve-omics.ce.gatech.edu/ani/; dDDH, http://ggdc.dsmz.de/distcalc2.php).

The *E. ictaluri* RUSVM-1 genome consists of one circular chromosome with 3,630,639 bp (57.4% G+C content). PGAP annotation predicted 3,377 genes encoding 3,254 proteins and 93 tRNAs. The RUSVM-1 genome is 5% smaller than that of *E. ictaluri* 93-146, isolated from catfish, which contains 3,783 predicted genes. RNAmmer (14) predicted 8 rRNA operons. RAST analysis predicted 484 subsystems with 3,643 coding sequences and 119 RNAs. The complete genome of RUSVM-1 has 99.5% ANI (dDDH, 96%) with* E. ictaluri* isolate 93-146 (15), 92.4% (dDDH, 48%) with *E. anguillarum* isolate LADL05-105 (16), 92.1% (dDDH, 48%) with *E. piscicida* isolate S11-285 (17), 83.0% (dDDH, 25%) with *E. tarda* isolate FL95-01 (18), and 82.5% (dDDH, 24%) with *E. hoshinae* isolate ATCC 35051 (19). Two plasmids were identified in RUSVM-1 and have been previously described (GenBank accession numbers KT937280 to KT937281) (20). This complete annotated genome sequence of *E. ictaluri* isolated from tilapia will be useful for future investigations into host-pathogen interactions and comparative *Edwardsiella* analyses.

### Accession number(s).

The complete genome sequence for *Edwardsiella ictaluri* isolate RUSVM-1 has been deposited in GenBank under the accession no. CP020466.
